# Establishment of an experimental ferret ocular hypertension model for the analysis of central visual pathway damage

**DOI:** 10.1038/srep06501

**Published:** 2014-10-13

**Authors:** Takashi Fujishiro, Hiroshi Kawasaki, Makoto Aihara, Tadashiro Saeki, Reiko Ymagishi, Takuya Atarashi, Chihiro Mayama, Makoto Araie

**Affiliations:** 1 Saitama Red Cross Hospital, Saitama, Japan; 2 Kanazawa University, Kanazawa, Japan; 3 Shirato Eye Clinic, Tokyo, Japan; 4 Koritsu Showa Hospital, Tokyo, Japan; 5 University of Tokyo School of Medicine, Tokyo, Japan; 6 Kanto Chuo Hospital, Tokyo, Japan

## Abstract

Glaucoma optic neuropathy (GON) is a condition where pathogenic intraocular pressure (IOP) results in axonal damage following retinal ganglion cell (RGC) death, and further results in secondary damage of the lateral geniculate nucleus (LGN). Therapeutic targets for glaucoma thus focus on both the LGN and RGC. However, the temporal and spatial patterns of degeneration and the mechanism of LGN damage have not been fully elucidated. Suitable and convenient ocular hypertension (OH) animal models with binocular vision comparable to that of monkeys are strongly needed. The ferret is relatively small mammal with binocular vision like humans – here we report on its suitability for investigating LGN. We developed a new method to elevate IOP by injection of cultured conjunctival cells into the anterior chamber to obstruct aqueous outflow. Histologically, cultured conjunctival cells successfully proliferated to occlude the angle, and IOP was elevated for 13 weeks after injection. Macroscopically, the size of the eye gradually expanded. Subsequent enlargement of optic nerve head cupping and atrophic damage of LGN projected from the OH eye were clearly observed by anterograde staining with cholera toxin B. We believe the ferret may be a promising OH model to investigate secondary degeneration of central nervous system including LGN.

Glaucoma optic neuropathy (GON) displays an axonal damage following retinal ganglion cell (RGC) death due to pathogenic intraocular pressure (IOP). Additionally, GON leads to secondary damage of central visual system processing vision via the optic nerve, i.e. lateral geniculate nucleus (LGN) and visual cortex (V1)^1^,^2^,^3^,^4^. Therefore, therapeutic targets for glaucoma focus on LGN and V1 as well as RGC. However, the temporal and spatial patterns of degeneration in the central visual system and the mechanism subsequently induced by GON have not been fully elucidated. Therefore, ocular hypertension (OH) models in which the central visual system is analyzed are strongly needed.

To date, many OH models utilizing rodents have been developed^5^,^6^,^7^,^8^,^9^,^10^,^11^,^12^,^13^,^14^,^15^. However, the visual system of these small animals is poorly developed compared to higher-order mammals because of nocturnal activity that is dependent on olfactory or auditory perception and poor binocular function. Thus, rodent models are not suitable for the analysis of the binocular central visual system. On the other hand, the monkey OH model is desirable because the relative time course of glaucoma development mirrors that of humans and the anatomical features of the ocular and central nervous system are similar to those of humans^16^,^17^,^18^,^19^,^20^. However, large numbers of experiments in monkeys have strong challenges. Thus, we selected ferrets to establish a desirable OH model because they possesses developed binocular vision compared to rodents^21^ and ferrets are relatively easy to breed compared to monkeys.

Ferrets are carnivorous mammals of the Mustelidae family and have a body of about 40 cm in length and eyeballs that are approximately 7 mm, and eyeballs in diameter are larger than those of rodents. The central nervous and visual systems are well developed^22^. Optic non-cross fibers are only 3–5% in the mouse, whereas they are approximately 15% in the ferret^21^,^23^,^24^. These features are desirable in the screening of neuroprotective drugs in the future. Therefore, ferrets have been accepted in Europe and the United States as experimental animals, and electrophysiological and morphological data in the ferret visual system have accumulated^25^,^26^,^27^. However, ferrets have not been used to study ophthalmic neuronal diseases such as glaucoma.

There is a report showing that conjunctival cells grow in the anterior cahmber, obstruct the trabecular meshwork and induce secondary glaucoma as known as epithelial downgrowth^28^. In this study, we developed a new, original method to elevate IOP in the ferret by injection of cultured conjunctival cells into the anterior chamber to obstruct aqueous outflow. Moreover, the subsequent changes caused by OH in the optic nerve and central visual system were histologically investigated.

## Results

Average IOP in the right eyes and left eyes during 13 weeks were measured. Next, the eyeballs were analyzed macroscopically and the eyeballs and optic nerve disc were analyzed histologically. Finally, the visual tract in the OH model was macroscopically and statistically analyzed using red and green cholera toxin B (CTB).

### IOP of OH ferret

Among 15 OH ferrets, IOP elevation was observed in 14 ferrets from the first week after cell injection. In one ferret, the right eye became infected 1 week after cell injection and the eyeball shrunk.

**Figure 1:**
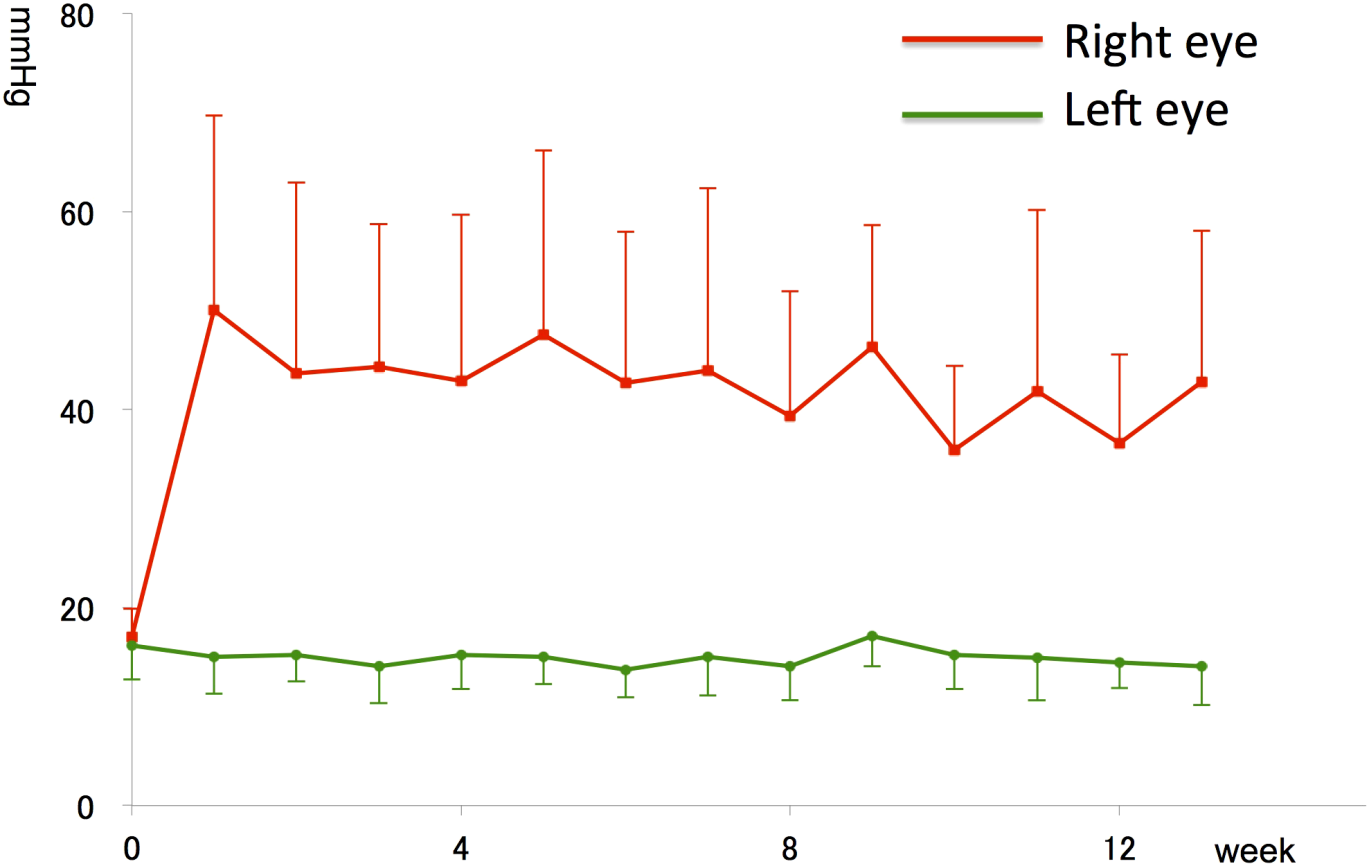
Intraocular pressure (IOP) in right (conjunctival cell-injected) and left (untreated) ferret eyes (mean IOP ± S.D.).

Average IOP in the right (treated) eyes and left (untreated) eyes during 13 weeks were 42.8 ± 15.3 (31–71) and 14.1 ± 3.9 (14–17) mmHg, respectively. IOP of treated eyes was significantly higher than IOP of untreated eyes (n = 14, paired t-test, p < 0.05) (Figure 1).

### Histological analysis of OH ferret model

#### Macroscopic changes of eyeball

**Figure 2:**
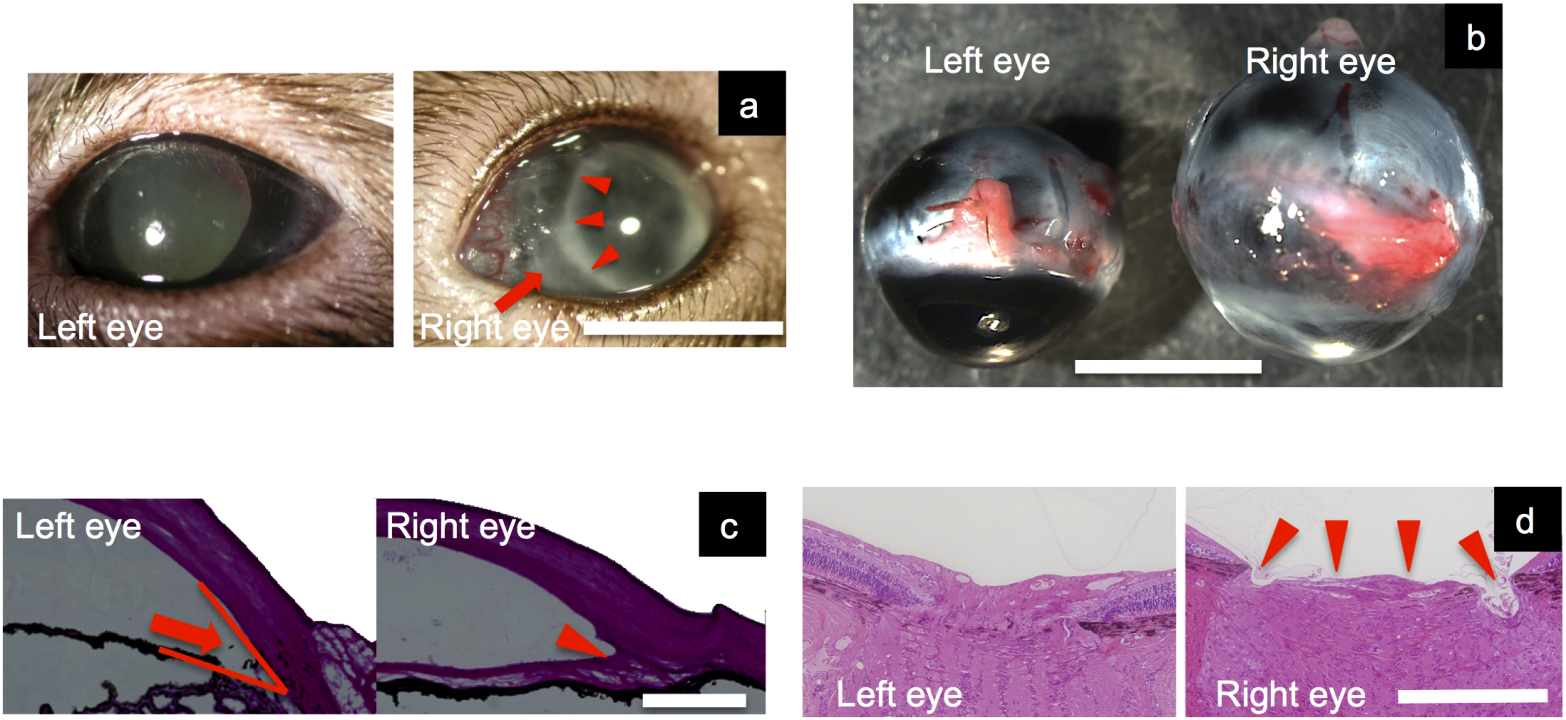
Comparison of an elevated IOP eye (right) and a normal eye (left), and meridian sections of H.E-stained eyeballs. (a) A photograph of an elevated IOP eye a week after conjunctival cell injection (right) and a normal eye (left). Proliferation of cells on the iris surface (arrow) and fibrous adhesion between the lens and iris (small arrows) were observed. Corneal edema was also seen due to high IOP. White bar is 5 mm. (b). Comparison of the diameter of the right (conjunctival cell-injected) and left (untreated) ferret eyes. Right (conjunctival cell-injected) ocular diameter was enlarged compared with left (untreated) ocular diameter. White bar is 5 mm. (c). Meridian sections of H.E-stained eyeballs (10 μm thick). Intraocular proliferation of injected cells (small arrows) had occurred and the angle was occluded. The arrow is the angle of the normal eye. White bar is 500 μm. (d). Optic disc cupping of right (conjunctival cell-injected) and left (untreated) ferret eyes. Enlargement of optic nerve head cupping (small arrows) was observed in H.E-stained sections. White bar is 500 μm.

Macroscopically, proliferation of cells on the iris surface and fibrous adhesion between the lens and iris were observed 1 week after injection. Corneal edema was also seen with high IOP (Figure 2a).

The size of the eye gradually expanded under the influence of high pressure. Thirteen weeks after the injection, the sizes of enucleated right (OH) and left (control) eyes were 7.76 ± 0.33 mm and 6.64 ± 0.13 mm, which was significantly different (n = 8, paired t-test, p < 0.01) (Figure 2b).

#### Histological analysis of eyeball

Histologically, intraocular proliferation of injected cells had occurred and the angle was occluded (Figure 2c). Enlargement of optic nerve head cupping was also observed (Figure 2d).

#### Histological analysis of optic nerve

**Figure 3:**
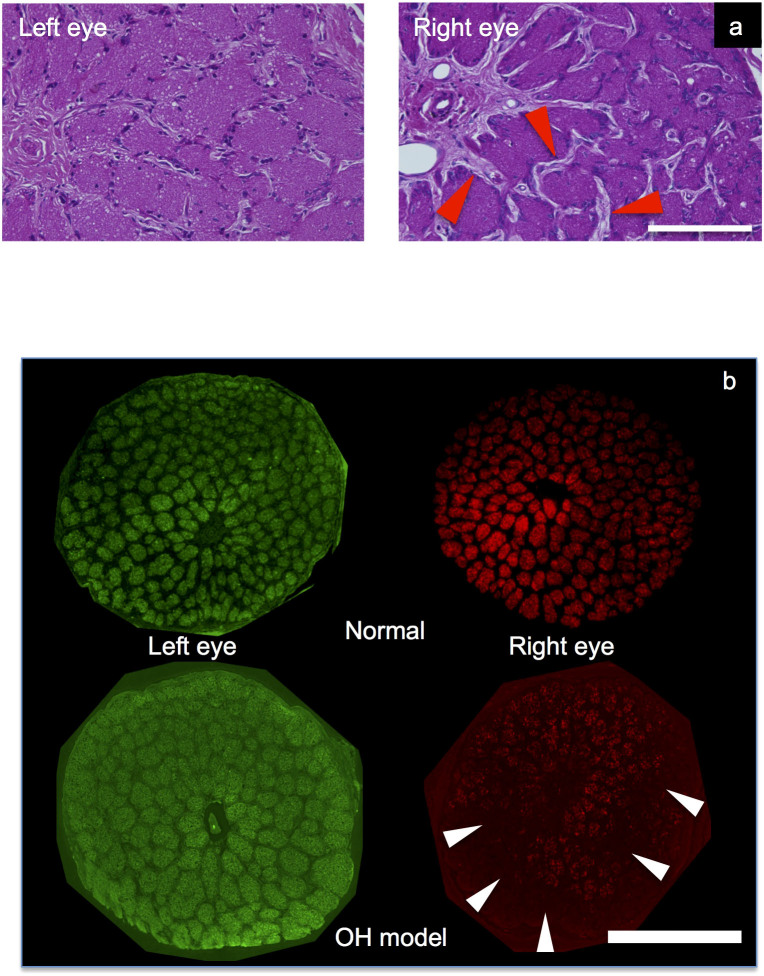
Microscopic images of cross sectional disc slices stained with H.E and CTBs. (a). Optical microscopic images of cross sectional disc slices stained with H.E at approximately 2 mm from the optic nerve head. Glial tissue was significantly increased in the right (OH) eye (small arrows) compared with the left (untreated) eye. White bar is 200 μm. (b). Cross-sectional disc slices at approximately 2 mm from the optic nerve head. The number of axon bundles of the right eyes of OH ferrets was significantly reduced compared with right eyes of bilaterally untreated ferrets. (small arrows) The number of axon bundles of the left eyes of OH ferrets was not significantly reduced compared with left eyes of bilaterally untreated ferrets. White bar is 500 μm.

In optic nerve cross sections, axon bundles surrounded by glial tissues were easily observed by optical and fluorescent images of axons stained by Hematoxylin-Eosin (H.E) and CTB. The axons were apparently reduced in the right optic nerve of the OH ferret compared to both the left optic nerve and optic nerve of the bilaterally untreated ferrets (Figure 3a, b).

The number of axon bundles of the right (OH) and left (control) eyes in the OH ferrets was 41.6 ± 25.8 and 132.6 ± 20.2. This represented a significant reduction in axon bundles in the right (OH) eyes (n = 5, unpaired t-test, p < 0.01) (Figure 3).

### Macroscopic analysis of the visual tract in the OH model

#### Macroscopic analysis of the visual tract

**Figure 4:**
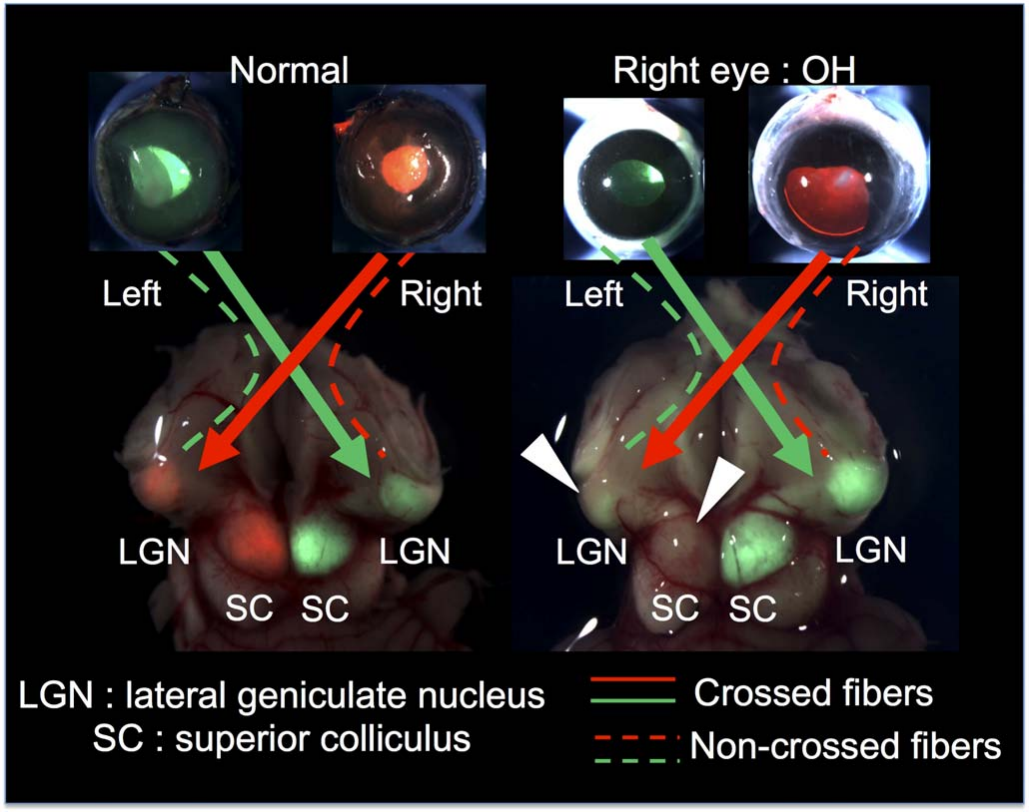
A photograph of the macroscopic visual tract in the OH ferret model. Red and green CTB injected into the right and left eyes, respectively, are projected on both sides of the superior colliculus (SC) and LGN in control ferrets. In the OH ferrets, red CTB from the right (OH) eye was not projected into the SC and LGN on both sides. (small arrows).

By injection of CTB into the eye, anterograde axonal transport was visualized and the damage of LGN was shown clearly. Before excision of the brain stem, CTB conjugated to Alexa 555 (red label) and CTB conjugated to Alexa 488 (green label) were injected into the right and left eye, respectively. Proper injection was macroscopically observed and confirmed by the fluorescence in the eyeball (Figure 4). In the bilaterally untreated ferrets, CTB conjugated to Alexa 555 (red label) was injected into the right eye and CTB conjugated to Alexa 488 (green label) was injected into the left eye were projected on both sides of the superior colliculus (SC) and LGN. In the OH ferrets, red CTB from the right (OH) eye was not projected into the SC and LGN on both sides (Figure 4).

#### Statistical analysis of the visual tract

The average value of brightness of OH ferrets was 37.4 ± 21.7 arb.unit in the right LGN (green CTB), and 9.1 ± 13.9 arb.unit in the left LGN (red CTB) (n = 8). The average value of brightness of control ferrets was 70.6 ± 8.1 arb.unit in the right LGN (green CTB), and 44.3 ± 11.0 arb.unit in the left LGN (red CTB) (n = 3). The average value of brightness in right and left LGN was significantly decreased in the right and left LGN of OH ferrets compared with right and left LGN of bilaterally untreated ferrets, respectively (unpaired t-test, p < 0.01).

## Discussion

This is the first report of the establishment of an OH model in ferrets. There are known difficulties in elevating IOP in this animal, and it was a big challenge to establish the OH model in ferrets. We tried previously established methods to increase IOP, such as episcleral vein occlusion^10^,^29^,^30^,^31^ and trabecular photocoagulation^32^,^33^,^34^, with no success. In addition, injection of latex beads^35^,^36^, viscoelastic materials^13^, and silicone oil^37^ was tried, but these injected materials were all extracted under the conjunctiva within a few days and IOP did not elevate. Next, we tried angle photocoagulation after intensive flattening of the anterior chamber by aspiration of aqueous fluid, similar to the method for OH mouse models^6^. However, in ferret eyes, the anterior chamber was immediately recovered and angle closure failed, probably due to the excessive production of aqueous fluid.

Finally, in reference to a report showing epithelial ingrowth leading to secondary glaucoma^28^, we tried an injection of a conjunctival cell suspension cultured *in vitro* and artificial angle closure was successful. Histologically, the angle was covered with proliferated cells as indicated in Figure 2c. One of the drawbacks of this method is that the fate and the mechanism of angle occlusion were not investigated in detail. To confirm the role of transplanted cells, we plan to transplant the allogeneic conjunctival fibroblasts with specific markers such as fluorescent protein.

There are only a few reports that measure IOP in ferret eyes^38^,^39^,^40^,^41^. IOP was measured by applanation tonometry (TonoPen® and TonoVet®) in conscious^38^,^39^,^40^ and anesthetized ferrets^41^. IOP varied from 14.50 ± 3.27 mmHg^38^ to 22.8 ± 5.5 mmHg^39^.

In this study, the IOP of control eyes was 17.36 ± 2.93 mmHg measured using a TonoLab® (Tiolat, Helsinki, Finland). Since IOP was determined in anesthetized ferrets, pharmacological interference of IOP may have occurred. Besides the potential influence of anesthetic procedures, several other factors could be responsible for IOP variations between studies including strain of ferrets, methods of physical restraint and version of the instrument measuring IOP^40^. Therefore, comparison of the present IOP values with previous values is not possible. However, the TonoLab® may be a better tool to measure IOP in ferrets because of the smaller standard deviation in the measured IOP value than those of previous reports.

In treated eyes, IOP significantly elevated to 31–71 mmHg, the eyeballs were expanded and optic disc cupping was enlarged (Figure 2d). In addition, 14/15 eyes showed over 100% IOP increase compared to untreated eyes. This rate was significantly higher than any other previously reported OH-inducing method in any animal species. Thus, this new method to induce OH may be applied to other animal species to increase IOP effectively and may achieve a higher success rate.

The IOP increase in this ferret OH model was sufficient to damage optic disc and axons in the eye. Optic nerve damage was characterized as degeneration of axons and thickening of the surrounding glial tissues in formed optic nerve bundles^8^. In this model, the area of optic nerve bundles and intake of fluorescein dye obviously decreased as indicated in Figure 3.

In addition to optic nerve degeneration, one advantage of the ferret OH model is that it may be suitable to investigate secondary degeneration of the optic tract accompanied with glaucomatous optic nerve degeneration in animals with binocular vision. As shown in monkey and human eyes, OH induced secondary degeneration of the LGN^4^,^42^,^43^,^44^. In this study, anterograde axonal transport was easily visualized by injection of CTB and this showed apparent damage in the optic tract of the right (OH) eye, and the damage was also statistically apparent in the optic tract of the left (untreated) eye. This result is coincident with the report that unilateral OH damage induces bilateral brain damage^44^. In the future, we plan to histological examine the secondary degeneration of LGN, SC and visual cortex in this ferret OH model as a representative small mammal with binocular vision.

There are some disadvantages of our ferret OH model. First, injection of conjunctival cells elicited intracameral inflammation, which may affect retinal degeneration. Second, the proliferative conjunctival cells prevented direct observation of the optic nerve and the retina. Thus, continuous *in vivo* imaging techniques cannot be applied for this OH model. Third, it was hard to regulate IOP to establish a mild glaucoma model such as human open angle glaucoma. The IOP values were similar to angle closure glaucoma and the IOP was so high that it could lead to ischemic optic neuropathy. However, high IOP inducing ischemia may be compensated by the expansion of eye globes as observed in buphthalmos of congenital glaucoma. In general, it has been a big challenge to establish an ideal glaucoma model similar to open angle glaucoma with only a mild increase in IOP without any inflammation or invasive tissue damage. Even the most popular OH mouse model, the DBA/2J strain, has inevitable degeneration of the anterior segment, cataract and some other ocular deficiencies^45^,^46^. In the future, we need to overcome these disadvantages of OH animal models.

In conclusion, injection of a cultured conjunctival cell suspension significantly increased IOP, induced optic disc cupping and degeneration of optic nerve axons in ferret eyes. Additionally, LGN and SC areas projected from the right (OH) eye were macroscopically damaged. In future studies, we plan to analyze the damaged area in the LGN or SC and to investigate the differences in the feasibility of axon damage among the subtypes of retinal ganglion cells, which is expected in the human eye with glaucoma.

## Methods

### Animals

The study was approved by the Research Ethics Committee of the Graduate School of Medicine and Faculty of Medicine at the University of Tokyo, and all animal experiments were performed in accordance with the Guidelines for the ARVO Statement for the Use of Animals in Ophthalmic and Vision Research.

Eighteen adult (16–32 weeks) female Marshall ferrets were obtained from Marshall BioResources (New York, U.S.A). The animals were housed in an environment of 23°C with a 12-hour light/12-hour dark cycle with free access to food and water.

### Anesthesia of ferrets

The ferrets were anesthetized by intramuscular administration of a mixture of ketamine (30 mg/kg) and xylazine (0.7 mg/kg) prepared at room temperature. Topical 0.4% oxybuprocaine (Benoxyl; Santen Pharmaceuticals, Osaka, Japan) was applied to both eyes before each treatment.

### Preparation of conjunctival cells

A 1 mm × 2 mm rectangle of conjunctival tissue was excised from a ferret, minced in phosphate buffered saline with 100 µg/ml streptomycin, and cultured under standard conditions (moist atmosphere, 5% CO_2_, 37°C) in Dulbecco's Minimum Essential Medium (DMEM) supplemented with 20% fetal bovine serum (FBS), and 100 µg/ml streptomycin for 8 weeks. At this time, only the conjunctival fibroblasts, which were determined morphologically, remain among the cultured conjunctival cells, and were fully confluent. Cultured fibroblasts were repeatedly cultured every 7 days after achieving full confluence.

### Injection of cultured cells and treatment of eyes

When the cells were approximately 90% confluent, the cells were trypsinized and cell suspension was made with DMEM with 20% FBS (3.3 × 10^4^ cell/ml; 50 μl). This cell suspension was injected into the anterior chamber of the right eye with a 32-gauge needle in ferrets (n = 15), and the left eye was untreated. After injection, the corneas of the injected right (OH) eye and left (untreated) eye were treated with an ointment of 0.3% ofloxacin. In addition, bilaterally untreated ferrets were also bred as control (n = 3). These ferrets were used for control in macroscopic analysis of the visual tract (figure 4).

### Measurement of IOP and ocular diameter

IOP was measured using the TonoLab® (Tiolat, Helsinki, Finland). Automatically averaged readings were recorded. When the statistical reliability of the average measurement, as represented by the coefficient of variance of the measurement, was not minimal, the reading was ignored and another measurement was taken. IOP was measured every week for 13 weeks after injection of the conjunctival cells in OH ferrets.

Ocular diameter was measured using a Digimatic Caliper Absolute™ (Mitsutoyo, Tochigi, Japan) at 13 weeks after conjunctival cell injection.

### Histological analysis of the eyeball and optic nerve disc in the OH model

After the ferrets were anesthetized, the eyes with intact optic nerve were enucleated. The eyeballs were quickly frozen with Optimal Cutting Temperature (OCT) compound (Sakura Finetechnical, Tokyo, Japan). Meridian sections of the eyeballs (10 μm thick) were then created and stained with H.E to evaluate disc cupping. Sections were observed using an optical microscope.

The optic nerves were separated from eyeball soon after enucleation. These were fixed with 3% glutaraldehyde and 2.5% formalin, and were frozen with OCT compound. Cross-sectional slices were created at approximately 2 mm from the optic nerve head and observed using an optical and a fluorescence microscope.

### Macroscopic and microscopic analysis of the visual tract in the OH model

To detect the connection and degeneration of the visual system from the eye to LGN, dyes were injected as described previously^27^,^47^. Briefly, 5 μl of CTB conjugated to Alexa 555 (red label) or conjugated to Alexa 488 (green label) was injected into the vitreous body with a 33-gauge needle at pars plana. Red and green CTB were injected into the right and left eyes of each ferret, respectively.

Eyeballs with optic nerves and the brain were removed 4 days after injection of CTB. To investigate the degeneration of the optic nerve, the number of axon bundles stained with red CTB in right eyes and green CTB in left eyes were microscopically counted and the exposure time was 0.5 second for each red and green CTB and compared. Next, the differences in staining of the brain stem were macroscopically observed in the OH and control ferrets using a fluorescence microscope. LGNs were also observed using a fluorescence microscope. The exposure time was 2.0 and 3.5 seconds for red and green stained LGN, respectively. The intensity of fluorescence in right and left LGN of OH ferrets were compared with right and left LGN of control ferrets. The average value of brightness of CTB fluorescence at the LGN was compared using Image J (U.S. National Institutes of Health, Bethesda, Maryland, USA).

### Statistics

All data are shown as mean ± standard deviation. The differences of IOP between injected eyes and control eyes were statistically evaluated by means of a paired t-test with Bonferroni correction. The difference of ocular diameter, number of axon bundles and the average value of brightness between injected eyes and control eyes were statistically evaluated by means of an unpaired t-test.

## Author Contributions

T.F., H.K., T.S., R.Y. and T.A. prepared the experiments. T.F. and M.A. wrote the main manuscript text prepared figures. H.K., M.A., C.H. and M.A. reviewed the manuscript.
